# The science of spin: targeted strategies to manufacture doubt with detrimental effects on environmental and public health

**DOI:** 10.1186/s12940-021-00723-0

**Published:** 2021-03-26

**Authors:** Rebecca F. Goldberg, Laura N. Vandenberg

**Affiliations:** 1 Graduate Program in Epidemiology, School of Public Health and Health Sciences, University of Massachusetts Amherst, Amherst, USA; 2 Department of Environmental Health Sciences, School of Public Health and Health Sciences, University of Massachusetts Amherst, 171C Goessmann, 686 N. Pleasant Street, Amherst, MA 01003 USA

**Keywords:** study design, hyperbole, regulation, logical fallacy, scientific literacy, misrepresentation, Tobacco Papers

## Abstract

**Background:**

Numerous groups, such as the tobacco industry, have deliberately altered and misrepresented knowable facts and empirical evidence to promote an agenda, often for monetary benefit, with consequences for environmental and public health. Previous research has explored cases individually, but none have conducted an in-depth comparison between cases. The purpose of this study was to compile a comprehensive list of tactics used by disparate groups and provide a framework for identifying further instances of manufactured doubt.

**Methods:**

We examined scholarly books, peer-reviewed articles, well-researched journalism pieces, and legal evidence related to five disparate industries and organizations selected for their destructive impacts on environmental and public health (tobacco, coal, and sugar industries, manufacturers of the pesticide Atrazine, and the Marshall Institute, an institute focused on climate change research, and other scientists from the era that associated with those in the Institute). These documents provided evidence for a list of tactics used to generate pro-industry spin and manufacture doubt about conferred harm. We then identified trends among sets of strategies that could explain their differential use or efficacy.

**Results:**

We recognized 28 unique tactics used to manufacture doubt. Five of these tactics were used by all five organizations, suggesting that they are key features of manufactured doubt. The intended audience influences the strategy used to misinform, and logical fallacies contribute to their efficacy.

**Conclusions:**

This list of tactics can be used by others to build a case that an industry or group is deliberately manipulating information associated with their actions or products. Improved scientific and rhetorical literacy could be used to render them less effective, depending on the audience targeted, and ultimately allow for the protection of both environmental health and public health more generally.

**Supplementary Information:**

The online version contains supplementary material available at 10.1186/s12940-021-00723-0.

## Background

The term ‘manufactured doubt’ refers to actions that deliberately alter and misrepresent knowable facts and empirical evidence to promote an agenda [1–4], often to benefit a broader industry, specific corporation, or group of individuals [1, 5]. The doctored, or spun, version of facts associated with manufactured doubt closely resembles the truth but is not easily discernible as falsehood [6]. Like an invasive species, it proliferates faster than the truth, spreading unchecked and able to adapt to specific constraints. Countless parties have used a suite of techniques and strategies to obscure the harmful effects of their work. This type of deceit can result in confusion among audiences, thus delaying actions that threaten the group’s mission and giving parties undue influence in the very systems intended to regulate them.

There are multiple examples of organizations that have manufactured doubt, obscuring the scientific link between their work/actions and harmful effects. These include, but are not limited to, the NFL and chronic traumatic encephalopathy (CTE), manufacturers of the insecticide DDT and wildlife destruction, pharmaceutical companies and the addictive nature of opioids, and asbestos companies and mesothelioma [1, 4, 7, 8]. These groups successfully spun a narrative predicated on manipulated facts, thus delaying environmental or public health protective actions, while calling the scientific basis for concern into question.

In a recent review, we described the deceptive actions of five different industries or organizations, chosen for their unique and varied contributions to the list of methods used to manufacture doubt among diverse audiences with ultimate impacts on environmental or public health [9]. The first, Big Tobacco, is widely considered to have “written the playbook” on manufactured doubt [1]. The *tobacco industry* has managed to maintain its clientele for many decades in part due to manufactured scientific controversy about the health effects of active and secondhand smoking [1, 2, 4, 6, 10–13].

The other industries we examined include *the coal industry*, whose employees often suffer from black lung disease [14], yet the industry has avoided awarding compensation to many affected miners by wielding disproportionate influence in the courtroom [15–19]; *the sugar industry,* which distracted from its role contributing to metabolic and cardiovascular diseases [20] by deflecting blame toward dietary fat as a plausible alternative cause for rising population-level chronic disease rates [21–25]; *the agrochemical business, Syngenta*, manufacturer of the herbicide atrazine [26–28], which conducted personal attacks against a vocal critic of atrazine whose research revealed disruptive effects on the endocrine systems of aquatic animals [29, 30]; and *the Marshall Institute*, a conservative think tank comprised of Cold War physicists eager to maintain their proximity to government, *and associated scientists* who deliberately misrepresented information to the government to both minimize and normalize the effects of fossil fuels on global temperatures [1, 4, 31].

The stories of these five industries and organizations reveal an extensive variety of tactics used to manufacture doubt within numerous organizations with impacts on environmental health and public health. A great deal of literature has examined these examples on a case-by-case basis, but to our knowledge, no evaluation has compared and contrasted these campaigns to identify common elements or strategies used across organizations to create pro-industry spin and manufacture doubt. In addition, to our knowledge, no prior work has provided a formal definition of manufactured doubt, though a close relative of the term is manufactured controversy, e.g., a controversy that is “motivated by profit or extreme ideology to intentionally create public confusion about an issue that is not in dispute” [32].

Here, we identify a list of tactics that were used by one or more groups to manufacture doubt. We describe each of these approaches and identify common trends that inform their use and efficacy. By distinguishing the breadth of tactics used by industries that impact public health, a definition of manufactured doubt was produced along with a framework that can be used to identify and characterize other instances in historical or emerging industries.

## Methods

### Searching the Literature & Identifying Tactics

We previously conducted a thorough review of literature concerning five doubt manufacturers: Big Tobacco, the coal industry, the sugar industry, Syngenta, and the Marshall Institute and associated scientists addressing climate change in the 1980s [9]. We selected these cases because each was already well-documented, and general knowledge of their intended audience or unique tactics was established within the field of environmental health. We also intentionally included a variety of organization types (e.g., single companies versus whole industries) to broaden the generalizability of our analysis. We also avoided specific companies with ongoing litigation at the time of our evaluation. We began this current analysis by examining the case of Big Tobacco, which may be the most widely recognized designer of strategies intended to obscure the harmful effects of its products (e.g. tobacco itself, active smoking, and secondhand smoking).

Starting with documents that are attributed to the Tobacco Industry (e.g., available on public databases including the University of California at San Francisco’s Tobacco Papers [33, 34]), and continuing with scholarly books, peer-reviewed journal articles, well-researched journalism pieces, and primary documents [33, 34], we searched for deceptive actions that demonstrated an attempt to falsify information, undermine facts, or spread misinformation. Methods with documentation of active as well as potential use by the industry were included. As additional tactics were identified, we listed and defined them in a table. We then repeated this task iteratively for the four other cases, examining additional scholarly resources and expanding tactic definitions to incorporate slight differences in use between cases. Source information is indicated in supplemental tables and reference lists.

### Organization and analysis of tactics

After fully investigating the five examples, we identified the strategies that were found in documents related to all five industries/organizations. We consider these common tactics to be the most effective tools for manufacturing doubt. We also identified the intended audience associated with each strategy. This information was organized into Venn diagrams to clearly demonstrate the shared or separate use of various techniques across audiences and industries.

Given the extensive literature relevant to these five case studies, we do not assume that our analysis is absolute; we may have overlooked evidence linking one or more strategies to one of the five organizations. There are no established criteria for identifying strategies so our decisions regarding which strategies to include, how to define them, and which were documented for each case study were dependent on our choice of literature and the breadth of literature available. In addition, determining the intent behind an organization’s actions is not always possible, and thus inferring whether an action involves willful deceit is dependent on our interpretation of the authors’ phrasing. We also did not conduct a systematic literature review, which was beyond the scope of this study.

## Results

**Fig. 1:**
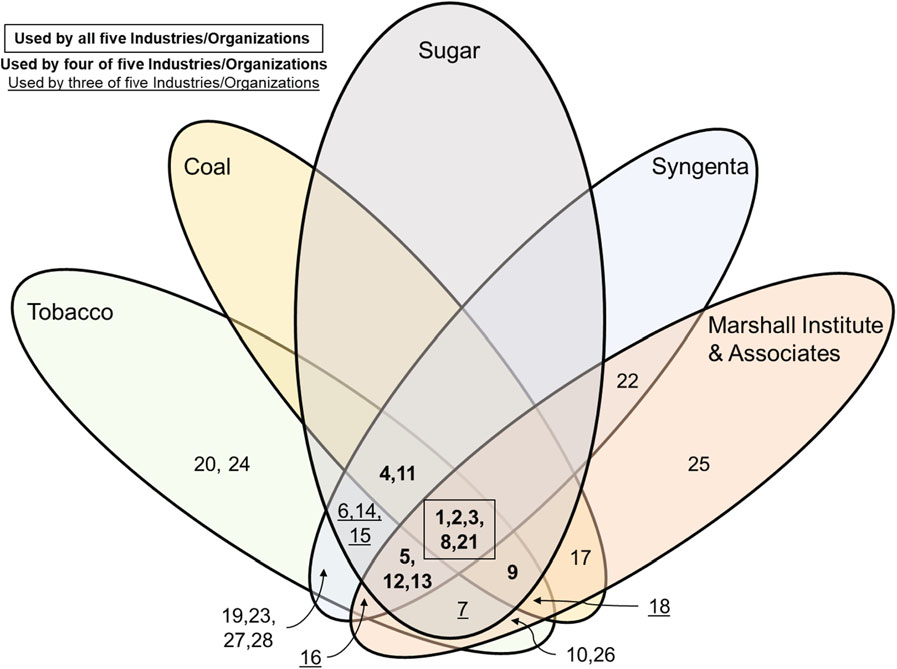
Strategies Utilized by the Five Industries/Organizations Examined. The placement of each strategy indicates which organizations were documented to use or planned to use, the indicated strategy. As indicated by the legend, strategies that are bold and outlined are shared by all five industries/organizations; strategies that are bold are shared by four industries/organizations; strategies that are underlined are shared by three industries/organizations. Strategies: #1, Attack Study Design; #2, Gain Support from Reputable Individuals; #3, Misrepresent Data; #4, Suppress Incriminating Information; #5, Contribute Misleading Literature; #6, Host Conferences or Seminars; #7, Avoid/Abuse Peer-Review; #8, Employ Hyperbolic or Absolutist Language; #9, Blame Other Causes; #10, Invoke Liberties/Censorship/Overregulation; #11, Define How to Measure Outcome/Exposure; #12, Take Advantage of Scientific Illiteracy; #13, Pose as a Defender of Health or Truth; #14, Obscure involvement; #15, Develop a PR Strategy; #16, Appeal to Mass Media; #17, Take Advantage of Victim’s Lack of Money/Influence; #18, Normalize Negative Outcomes; #19, Impede Government Regulation; #20, Alter Product to Seem Healthier; #21, Influence Government/Laws; #22, Attack Opponents; #23, Appeal to Emotion; #24, Inappropriately Question Causality; #25, Make Straw Man Arguments; #26, Abuse Credentials; #27, Abuse Data Access Requests; #28, Claim Slippery Slope

We identified 28 unique strategies (Table 1) used by organizations either to combat scientific evidence and facts (referred to in the table as Information A) or to promote narratives that are favorable to the industry (referred to in the table as Information B) (Table 1). Five of these strategies were used by all five organizations (Fig. 1): attacking study design used to produce Information A (#1), gaining support from reputable individuals to defend Information B (#2), misrepresentation of Information A (#3), employing hyperbolic language (#8), and influencing government agencies or laws (#21). We argue that these five strategies are the most effective features of manufactured doubt (i.e., highly successful at delivering a message to an intended audience) and together provide the strongest indication that an industry is communicating manufactured rather than authentic doubt. The absence of any of these features, however, should not be interpreted as the failure to build a case for manufactured doubt. See Additional File 1 for more detailed references for each of the five cases.

**Table 1:** List of Strategies Used by Five Industries/Organizations to Manufacture Doubt

	Strategy	Explanation	Tobacco	Coal	Sugar	Syngenta	Marshall Institute & Others
1	Attack Study Design	To emphasize study design flaws in A^ϕ^ that have only minimal effects on outcomes. Flaws include issues related to bias, confounding, or sample size	X	X	X	X	X
2	Gain Support from Reputable Individuals	Recruit experts or influential people in certain fields (politicians, industry, journals, doctors, scientists, health officials) to defend B^Δ^ in order to gain broader support	X	X	X	X	X
3	Misrepresent Data	Cherry-pick data, design studies to fail, or conduct meta-analyses to dilute the work of A	X	X	X	X	X
4	Suppress Incriminating Information	Hide information that runs counter to B	X	X	X	X	
5	Contribute Misleading Literature	Use literature published in journals or the media to deliberately misinform, either pro-B, anti-A, or to distract with peripheral topics	X		X	X	X
6	Host Conferences or Seminars	Organize conferences for scientists or relevant stakeholders to provide a space for dissemination of only pro-B information	X		X	X	
7	Avoid/Abuse Peer-Review	Avoid the peer-review process to publish poor literature, publish without revealing funding sources, use the journal name to add weight to claims, or minimize need for peer-review among lay audiences	X		X		X
8	Employ Hyperbolic or Absolutist Language	Discuss scientific findings in absolutist terms or with hyperbole, use buzzwords to differentiate between “strong” and “poor” science (i.e. sound science, junk science, etc.),	X	X	X	X	X
9	Blame Other Causes	Find related, alternative causes for negative effects that are reported or observed	X	X	X		X
10	Invoke Liberties/Censorship/ Overregulation	Invoke laws to emphasize equality and rights for expression of B, despite differences in evidence quality	X				X
11	Define How to Measure Outcome/Exposure	Attempt to set guidelines for ‘proper’ measurement of exposures or outcomes, while undermining guidelines used in A	X	X	X	X	
12	Take Advantage of Scientific Illiteracy (media/individuals)	Emphasize scientific obscurity to confuse lay audiences, or deliberately disseminate unscientific or false but digestible information	X		X	X	X
13	Pose as a Defender of Health or Truth	Represent the goals of B as health-conscious or dedicated to truth	X		X	X	X
14	Obscure involvement	Ghostwrite, create shell companies, use attorney client privilege to hide association	X		X	X	
15	Develop a PR Strategy	Devise methods for specifically reaching public audiences to spread B messages	X		X	X	
16	Appeal to Mass Media	Appealing to journalistic balance, developing relationships with media personnel, preparing information for media personnel, invoking the Fairness Doctrine	X			X	X
17	Take Advantage of Victim’s Lack of Money/Influence	Silence or abuse individuals by out-spending or exploiting a power imbalance		X			X
18	Normalize Negative Outcomes	Normalize the presence of negative effects to reduce importance and make them seem inevitable	X	X			X
19	Impede Government Regulation	Overwhelm governmental regulatory agencies to slow or stop their function	X			X	
20	Alter Product to Seem Healthier	Make modifications to harmful product to reduce ostensible negative effects	X				
21	Influence Government/Laws	Gain inappropriate proximity to regulatory bodies and encourage pro-B policy	X	X	X	X	X
22	Attack Opponents (scientifically/personally)	Conduct targeted attacks on opponents by undermining their professional or personal reputations				X	X
23	Appeal to Emotion	Manipulate an audiences’ emotions to draw support for claims in the absence of facts	X			X	
24	Inappropriately Question Causality	Argue that correlation does not equal causation despite the presence of strong evidence	X				
25	Make Straw Man Arguments	Publicly refute an argument that was not made by the opposition					X
26	Abuse Credentials	Use qualifications in one discipline to assume authority in another discipline	X				X
27	Abuse Data Access Requests	Requesting access to data in order to misrepresent and attack, employing Shelby Amendment, Freedom of Information Act, etc..	X			X	
28	Claim Slippery Slope	Illogically or falsely claiming that there will be disastrous consequences if B ideology is not supported	X			X	

^ϕ^ “A” refers to information generated to combat scientific evidence and facts

^Δ^ “B” refers to information generated to promote narratives that are favorable to the industry

Many other tactics were used by four of the five organizations that we evaluated, suggesting that they are individually effective though not consistently used to manufacture doubt; their presence adds to the weight of evidence that a group is manufacturing doubt. This set of tactics also suggests that there may be aspects of the industry, product, or intended audience, that dictate the actions taken or not taken by a specific industry.

Only three strategies were used by a single industry each: the tobacco industry altered their product to make it appear safer (#20); the tobacco industry inappropriately questioned causality (#24); and prominent climate denialist scientists from the 80’s created straw man arguments to combat action against climate change (#25). Of note, several strategies utilized by one or more industry or organization rely on logical fallacies (Table 2), a well-established form of rhetorical manipulation.

**Table 2:** Logical Fallacies in Strategies to Manufacture Doubt

	Strategy	Logical Fallacy
2	Gain Support from Reputable Individuals	Appeal to authority (*ad vercundiam)*: saying that because an “authority” believes something, it must be true
3	Misrepresent Data	Texas Sharpshooter: utilizing a subset of evidence that supports a theory but ignoring the full picture
9	Blame Other Causes	Questionable Cause (*cum* *hoc ergo propter hoc*): confusing correlation with causation
11	Define How to Measure Outcome/Exposure	Definist Fallacy: redefine a term to make a position easier to argue
13	Pose as a Defender of Health or Truth	Righteousness Fallacy: using evidence of good intentions to support other claims
22	Attack Opponents	*Ad hominem*: by attacking the arguer instead of the argument, the argument can be dismissed
23	Appeal to Emotion	Appealing to emotion: manipulating an emotional response in place of a valid, factual, compelling argument
25	Make Straw Man Arguments	Strawman argument: Misrepresenting an argument to make it easier to attack
26	Abuse Credentials	Use of false authority: using an expert with dubious or unrelated credentials to promote the industry’s position
28	Claim Slippery Slope	Slippery Slope: avoiding the main argument by using extreme hypotheticals as distractions

To be effective, manufactured doubt requires that a message, predicated on altered evidence, reach its intended audience. We identified four different audiences that are typically targets of manufactured doubt: the scientific/medical community, political organizations including government officials, the lay public, and the judicial/legal system. In our analysis of the tactics described above, we found that in many cases the intended audience drives the process, and distinct audiences require different types of (dis)information.

**Fig. 2:**
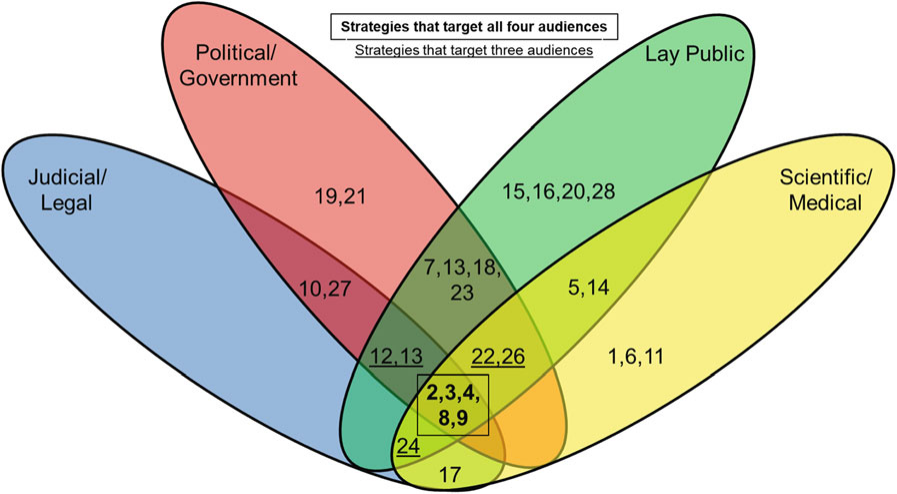
Audiences Targeted by the Specific Strategies Utilized to Manufacture Doubt. The placement of each strategy indicates which audience(s) were targeted by the indicated strategy. As noted by the legend, strategies that are bold and outlined are used to target all four audiences; strategies that are underlined are used to target three of the four audiences. Strategies: #1, Attack Study Design; #2, Gain Support from Reputable Individuals; #3, Misrepresent Data; #4, Suppress Incriminating Information; #5, Contribute Misleading Literature; #6, Host Conferences or Seminars; #7, Avoid/Abuse Peer-Review; #8, Employ Hyperbolic or Absolutist Language; #9, Blame Other Causes; #10, Invoke Liberties/Censorship/Overregulation; #11, Define How to Measure Outcome/Exposure; #12, Take Advantage of Scientific Illiteracy; #13, Pose as a Defender of Health or Truth; #14, Obscure involvement; #15, Develop a PR Strategy; #16, Appeal to Mass Media; #17, Take Advantage of Victim’s Lack of Money/Influence; #18, Normalize Negative Outcomes; #19, Impede Government Regulation; #20, Alter Product to Seem Healthier; #21, Influence Government/Laws; #22, Attack Opponents; #23, Appeal to Emotion; #24, Inappropriately Question Causality; #25, Make Straw Man Arguments; #26, Abuse Credentials; #27, Abuse Data Access Requests; #28, Claim Slippery Slope

We identified five strategies that can be used regardless of the intended audience (Fig. 2): gain support from reputable individuals (#2), misrepresent data (#3), suppress incriminating information (#4), employ hyperbolic language (#8), and blame other causes (#9). These strategies were used by at least four of the groups we examined (Fig. 1); three of them (#2, #3, and #8) were used by all five groups, suggesting that they are especially effective because they can target all relevant audiences.

Many other strategies target only three of the audiences, but due to their nature are unlikely to be effective at altering perceptions in the fourth audience. For example, taking advantage of scientific illiteracy and posing as a defender of health or truth likely will not be useful in the scientific community specifically because literacy and objectivity are core tenets of scientific inquiry. Similarly, attacking opponents and abusing credentials are unlikely to hold weight in the legal community because the legal system prevents personal attacks (i.e., arguments must be predicated on law, not public opinion) and expert witnesses can only serve after passing evaluation as an expert in their field. A few strategies are singular to an audience. For example, appealing to mass media (#16) is aimed toward a general lay audience. Scientists, government officials, and the court system rely on other sources of information for decision making. Attacking study design (#1) is aimed at the scientific community, where a basic understanding of study design and bias are needed to understand both a paper and its criticism.

## Discussion

We have identified a list of 28 unique tactics used in five diverse case-studies to manufacture doubt. Five of these strategies were used by all five organizations we examined, suggesting that they are sufficient for the protection of products, to the detriment of public health. Based on all information gathered herein, we have created a working definition for manufactured doubt: the deliberate alteration and misrepresentation of knowable facts and empirical evidence to promote an agenda, often for financial benefit.

Many of these strategies require funds for implementation. For example, tactics such as the recruitment of reputable individuals require monetary resources as these in-demand professionals typically expect to be compensated for their time and/or efforts; contributing to the published literature can be costly if ghostwriters or professional scientists are hired; hosting conferences and seminars can require funds to pay for travel costs, honoraria for reputable speakers, and venue fees; and the use of professional firms to develop PR campaigns can require significant funds depending on the nature of the work. Other strategies take advantage of a victim’s lack of money or influence, which necessarily requires vast amounts of resources to maintain a power imbalance. For example, the coal industry’s victims - its own employees - often have limited funds, scientific and medical knowledge, or political and social capital.

The industries and organizations involved in all five of the case studies we selected had both money and influence at their disposal. The coal, sugar, and tobacco industries are composed of multiple large companies that possess the funds needed to purchase support and attack opponents. Syngenta is one of the largest agrochemical companies operating worldwide. The Marshall Institute received private funding, often from fossil fuel shell companies or think tanks, to continue its work and held sway over governmental opinions and policies, thus it did not need to purchase access [35]. Funding is not only a necessary component to support many of the strategies to manufacture doubt, all five groups also financially benefitted from their ability to manufacture doubt. Thus, financial power is an essential component of manufactured doubt.

The true reasons for the common use of five strategies (#1, #2, #3, #8, #21) among all five case-studies is unclear, though our analysis of the intended audience provides some insight. Strategies #2, #3, and #8 target all audiences (Fig. 2), indicating they effectively spread (dis)information. Other strategies that target all audiences but were not universally employed in the five case-studies (strategies #4 & #9) were documented in 4 of the 5 groups we evaluated, further supporting that a ubiquitous audience contributes to the efficacy of an approach used to manufacture doubt. Furthermore, the absence of use of these strategies in the fifth case-study might be attributed to lack of documentation in the literature, or there may be a specific reason why these strategies were not effective in these specific cases.

We also found that many of the tactics used to manufacture doubt rely on logical fallacies to misrepresent information. For example, abusing credentials (#26) is a form of appealing to a false authority; individuals with expertise in one field are routinely recruited to support industry actions in unrelated fields, and their reputations as experts are exploited to promote industry’s viewpoint to the detriment of truth and health. An illustrative example of this logical fallacy comes from disgraced physician Andrew Wakefield. Wakefield was a gastroenterologist who claimed to have found an association between use of the vaccine for measles, mumps and rubella and autism in children [36]. Not only did Wakefield lack training in virology or neurodevelopmental disorders, evidence later demonstrated that he intended to capitalize on the anti-vaccine movement through development and marketing of molecular diagnostic tests which he had patented [37]. He continues to promote anti-vaccination ideology, invoking several logical fallacies including the use of slippery slope arguments, to gain personal support but with great threats to public health [38].

In an increasingly interdisciplinary world, identifying logical fallacies may become an essential part of scientific training. Learning to identify their use requires “training in critical thinking and avoiding illogical thought patterns that often come naturally to humans” [39]. Even well-trained scientists can perform poorly on tests evaluating “straightforward” logical relationships [40]. For this reason, lay audiences, who often lack formal training in critical and logical thinking, may find the process of identifying and rejecting the logical fallacies used to manufacture doubt particularly difficult. Logical fallacies have been discussed in the context of numerous and disparate scientific fields and are not always observed in the context of manufactured doubt (e.g., [41, 42]); rather, they may solely reflect the communicator’s own logical reasoning. Thus, the presence of a logical fallacy is insufficient to conclude that a group or individual is intending to manufacture doubt.

We believe our analysis of the tactics used by industries and organizations can also be used to answer the question of *who* can manufacture doubt. Our approach provides evidence that there are two related but distinct parties in the doubt manufacturing process: manufacturers and perpetuators. All five of the groups we examined worked with others such as PR firms, scientists, researchers and physicians, as well as hired “ringers” posing as lay-people, who served as active doubt manufacturers (e.g., those who directly manipulated information, actively formulating, or employing one of the 28 listed strategies) ([43], pg 73).

In contrast, doubt perpetuators knowingly or unknowingly spread false information given to them by an original doubt manufacturer. These can include, but are not limited to, journalists, bloggers, citizen scientists, and lay-people that disseminate information and spread pro-industry spin. For example, one member of the Marshall Institute found that the Wall Street Journal [44] served as a suitable platform for circulating a pithy attack on the IPCC [3]. Scientific information can be complex, and communicating such information often requires lengthy analysis or explanation; thus, it is often poorly suited to popular methods of communication which have become increasingly brief and reliant upon common knowledge [45]. Journalists, perhaps even recognizing falsehoods, face challenges in exposing manufactured doubt to the public when it runs counter to public ideology, and unfortunately many media platforms do not allow for in-depth analyses. As the cases described here demonstrate, lengthy journalism pieces that reveal a strong case of manufactured doubt can result in a paradigm shift, forcing us to reanalyze sometimes decades-old assumptions; in contrast, a short article or two-minute news segment are likely insufficient for communicating the methods and implications of an entire case. Despite these limitations, investigative journalism has played an extremely important role in uncovering instances of manufactured doubt (including several of the case-studies described here). Resources such as First Draft, an international organization that aims to train journalists and arm the public to identify and understand disinformation, are becoming available to aid in dissemination of complex, evidence-based information.

The digital age has provided additional opportunities to spread misinformation. Doubt manufacturers have taken advantage of new media platforms, such as blogs and social media, to unite journalists, industry representatives and ‘citizen scientists’ with the aim of recruiting these individuals to perpetuate manipulated information ([43], pg 70), [46]. These methods of dissemination exist in addition to print, video, and radio methods already heavily drawn upon to spread doubt. The internet provides a unique platform for individuals and organizations that aim to manufacture doubt, allowing them to reach an enormous audience without investment of time or funds. In the interest of free speech, social media platforms specifically have delayed in enacting fact-checking policies [47], with the unintended effect of allowing disinformation to spread.

The average individual can be easily swayed to denial, perhaps due to confirmation bias stemming from ideological, political, or religious beliefs or a lack of subject-specific knowledge for critically examining scientific evidence. Thus, the general public may participate in “muddying the waters” by acting as unknowing recipients and perpetuators of manufactured doubt. When manufacturing doubt, industries capitalize on grassroots support, encouraging citizens to take part in actions that impede public health-promoting activities and spread disinformation among family, friends, and relevant parties ([43], pg 71). The amount of pseudoscientific and unfounded health-related information provided to the public and shared by the public around the globe in our current times, such as through social media, has only proven the absolute danger of this phenomenon, where misrepresented information results in lost lives [48, 49].

Of note, in the case studies we examined, governmental bodies were not doubt manufacturers; instead, outside industries influenced federal government agencies to become unknowing doubt perpetuators. Affiliated groups (such as the Marshall Institute) or lobbyists working for industries provided information to federal agencies, and, unfortunately, members of those agencies could not always sort truth from lies. For example, the Marshall Institute presented a report to members of the federal government that blamed the sun for increasing global temperatures; in this report, they selectively displayed data visualizations that supported their claims and omitted those that ran counter [50]. The report was well-received in the White House, where cabinet members praised its merits. A representative reported that “policy in the federal government … is not inconsistent with the Marshall Institute report” [51]. There are multiple stopgaps in a democratic society that hinder the spread of misinformation from and within all levels of government, including bureaucratic oversight and an active free press. Access to public records and investigative journalism can prevent governments from making financial investments and enacting policies that confuse or harm the public (at least when the public and other oversight bodies are also able to discern truth from falsehood). Private individuals and institutions have none of the same oversight, and their immense number can also inhibit widespread investigations.

Examples where government employees and elected officials have perpetuated doubt suggest that even these individuals with great power can fall victim to the same scientific illiteracy and logical fallacies that influence the general public. Further, employees in many federal agencies benefit from the “revolving door” between industries and the agencies tasked with their regulation, leading these employees to favor decisions that benefit industries (i.e., their future employers) [52]. Thus, the same policies that would impede manufactured doubt are subject to influence from doubt manufacturers.

Of note, evidence from other cases not studied here demonstrates that government can also be a manufacturer, not just a perpetrator, of doubt. For example, the US federal government (and more specifically, the Department of Defense) contaminated drinking water with perchlorate, a chemical used in missiles and rockets that interferes with iodine uptake [53]. Studies suggest that US regulatory agencies erred in determining the amount of perchlorate that damages development of the fetal brain (e.g., even very low exposures to perchlorate, equivalent to daily intake of ~ 0.5 μg/kg/day [54], are associated with lower IQ, contrary to federal regulatory standards) [55, 56]. In this case, the federal government had a vested interest in producing and promoting disinformation about a public health hazard that it was responsible for creating. In other examples, Chomsky and others argue that the US federal government has manufactured doubt, with assistance from the media, about the true rationale and justification for conflicts and wars the US has engaged in for several decades [57]. In both of these examples, the federal government manipulated the public’s knowledge about actions that led to loss of human life, diminished human health, and other devastating effects. A governmental body acting as either a manufacturer or perpetuator of doubt, depending on the circumstances, risks more than just lives lost from that one case; it also undermines any future attempts by the government at making evidence-based decisions and damages the public’s trust [58].

We propose that our list of 28 tactics can be used as a tool for both identifying and countering new cases of manufactured doubt as they arise in emerging industries with obvious implications for environmental health, and public health more broadly (e.g., e-cigarettes, nanotechnology, fracking) [59–61]. Similarly, this list can be applied to documents and information obtained regarding “old” industries as new concerns are raised (e.g., the safety of chlorpyrifos or glyphosate, pesticides that continue to be defended by agrochemical companies [62, 63]; the over-prescription of opioids and denial of their addictive nature by the pharmaceutical industry [64]); and even COVID-19 (and the promotion of treatments such as hydroxychloroquine, which research shows to be ineffective while posing risks to patients) [65]. When an organization alters and misrepresents empirical evidence to promote an agenda, then a search for the use of further tactics is warranted. Evaluations of these old and emerging industries will be limited by the availability of documentation and other supporting evidence; the legal discovery process has been essential to building the five case studies we examined and building a case for manufactured doubt without such documents would be challenging.

We note an important caveat regarding this type of evaluation. Some of the 28 tactics we identified can be used for legitimate purposes, so these tactics must be evaluated in a broader context. For example, public health defendants with strong evidence may err but nonetheless choose to use hyperbole. Advocates for environmental health issues may host one-sided conferences without the intention to misinform; of course, there is no need for legitimate scientific organizations to invite speakers representing a non-scientific viewpoint to their conferences in an attempt to avoid appearing biased. Additionally, anyone is capable of unintentionally employing a logical fallacy, and as noted above, the use of logical fallacies alone is not evidence that a group is manufacturing doubt. Thus, expert knowledge or informed judgment must be used when applying this analysis to other cases.

Furthermore, the use of each of these strategies alone does not necessarily indicate deception; we suggest that both the strength and weight of evidence must be considered. The use of a variety of strategies provides stronger evidence of an agenda to manufacture doubt. Similarly, the use of a weightier strategy, such as suppressing information, compared to one that may be used for non-manipulative purposes, such as hosting conferences, also provides more compelling evidence for manufactured doubt. For this reason, the 28 strategies should not be considered to have equal weight. As new cases are evaluated, we would expect that the five strategies that were common to the case studies we examined here (#1, #2, #3, #8, #21) would similarly be documented; such observation is likely to provide the most convincing evidence for manufactured doubt.

Ultimately the responsibility for the production of disinformation, and its effects on individuals, public health, and the loss of public trust lies with the doubt manufacturers. Until principles of scientific integrity are incorporated into the mission statements of all industries, organizations, and government agencies, recipients of information must be better prepared to identify falsehoods. Policies must also be specifically designed by government agencies, academic institutions, scientific and medical societies, and other stakeholders to stop its spread. Furthermore, the ramifications of regulatory “reform” legislation intended to stem the creation of doubt, or the spread of doubt, should be made clear to constituents who may be unaware of its impact on evidence-based policy [66]. Defining manufactured doubt and identifying the methods for its implementation are necessary for developing strategies to counter its effects and prevent propagation. Manufactured doubt continues to invade scientific, social, political, and legal spheres; therefore, diverse audiences must develop skills to recognize it and the methods used to generate it. Identification is a difficult task when the results of manufactured doubt inherently allow invested parties to remain hidden. By determining the intended audience of each strategy, we are better poised to identify specific methods of countering their effects. There are a variety of strategies that individuals can use to evaluate and contextualize the information they are given, but they differ by audience:

1. The lay public can verify sources and investigate who is making claims by checking for conflicts of interest. Though public health may not motivate all individuals, and not everyone is interested in overcoming confirmation bias, knowledge of potential threats to personal health through intentional deception may inspire more thorough personal investigation. Enhanced understanding of logical fallacies would also increase the public’s recognition of their use. Media must also avoid presenting evidence and industry-generated manufactured doubt or “spin” as if these two views are equal. As shown in these cases, this kind of “balance” does not reflect truth.
2. Scientists, who have a greater understanding of the evidence-based literature, must also identify conflicts of interest such as funding, consider the credentials of “experts”, and be wary of meta-analyses or attacks on scientific articles by consulting original studies. Peer-reviewed journals have an obligation to require conflict of interest disclosure and should refuse papers that are designed to mislead. Lack of disclosure should be followed by the retraction of studies. Research funding from big businesses may not necessarily indicate bias, but reviewers must be trained to consider bias when a relationship is disclosed. Scientists also benefit from positions of power within organizations (e.g., academic institutions, medical and scientific societies). They should utilize their roles to advocate for change within these institutions, which will help to improve the public’s access to scientific information [67].
3. In the legal system, there is much room for improvement regarding equity. Policies can be enacted that protect the rights of individuals affected by industry’s actions (like coal miners) to effectively defend their cases, without the influence of a power or financial disparity. Expert testimony must also be held to a very high standard of impartiality.
4. The current political system has demonstrated that funding influences regulations and policy. Though we cannot expect all government officials to be experts in scientific literacy, government agencies must actively consult and act upon information from truly impartial individuals that can provide support and information regarding scientific and non-scientific evidence not subject to manufactured doubt. We can ask that the executive branch nominate qualified individuals to lead scientific agencies and advisory boards; that federal officials face punishment for tampering with scientific reports; and that administrations, regardless of their political party, avoid creating hostile work environments for scientific staff [68]. Regulatory agencies must also acknowledge and stop the revolving door with industry and take actions to protect against biases that are introduced by future employment relationships.

Individuals and organizations, especially those tasked with protecting the public’s health, must arm themselves with knowledge of these tactics and develop or hone protective strategies to recognize and prevent cases of manufactured doubt. This investigation will enable people from all spheres to assess the vast amount of information they are exposed to in the most objective way possible and discern truth from falsehood.

Our evaluation provides a thorough, but by no means exhaustive, list of tactics used by industries and organizations to manufacture doubt. Our list is limited by the scope of the works cited, the documentation available, and the current understanding of stakeholders involved in these cases; there may have been strategies that were used but not mentioned in the covered texts, and information regarding the covert actions taken by manufacturers may not yet be available to the public. Null findings may indicate a true absence of use or the limitations of publicly available documentation. We also make no assessments of the scientific literature involved in these cases (e.g., our intent is not to demonstrate that tobacco itself is hazardous to health); rather we have focused this research on efforts made by organizations to misrepresent evidence by any means and the possible repercussions of those falsified narratives.

The conclusions and definitions reached in this analysis may be specific to the groups we investigated. Our list is also defined by the scope of the case studies selected (i.e. a specific company vs. an industry), and a more inclusive case-study may contribute a wider range of tactics. With more cases, an even more refined definition of manufactured doubt can be produced. Future analyses could include instances of government-sanctioned manufactured doubt or more recent cases. The methods described herein, however, are integral to understanding industry ‘spin’ and warn against the implications of as-of-yet undiscovered tactics and doubt mongering.

## Supplementary Information

**Additional file 1.**
