# Supplementary material for: The science of spin: targeted strategies to manufacture doubt with detrimental effects on environmental and public health
Source: Environ Health. 2021 Mar 26;20:33. doi: 10.1186/s12940-021-00723-0 (PMC7996119; doi:10.1186/s12940-021-00723-0)
Supplement: Supplementary file 1 — Additional file 1. [file 12940_2021_723_MOESM1_ESM.docx]

Supplemental Table 1. List of Strategies Used by the Tobacco Industry and Affiliates

| # | Strategy | X = used | Evidence Available From: |
| --- | --- | --- | --- |
| 1 | Attack Study Design | X | Unknown Author. PROPOSAL TO STUDY INTERVIEWER BIAS. 1997 May. Philip Morris Records. Available from: <https://www.industrydocuments.ucsf.edu/docs/flwc0088>. Accessed 01 December 2019. |
| 2 | Gain Support from Reputable Individuals | X | SIRRIDGE, PM. Tobacco Industry Research Committee - Council for Tobacco Research [Privlog:] DRAFT SPEECH FROM PHILIP MORRIS COUNSEL TO PHILIP MORRIS COUNSEL REGARDING TOBACCO INSTITUTE. Unknown date. Philip Morris Records; Congressman Bliley Philip Morris Collection. Available from: <https://www.industrydocuments.ucsf.edu/docs/hyby0101> Accessed 01 December 2019.  Cited in:  Oreskes, N., & Conway, E. M. (2010). *Merchants of doubt: How a handful of scientists obscured the truth on issues from tobacco smoke to global warming*. New York: Bloomsbury Press. |
| 3 | Misrepresent Data | X | Wertz MS, Kyriss T, Paranjape S, Glantz SA. (2011) The Toxic Effects of Cigarette Additives. Philip Morris' Project Mix Reconsidered: An Analysis of Documents Released through Litigation. PLoS Med 8(12): e1001145. |
| 4 | Suppress Incriminating Information | X | Unknown Author. Trial testimony of JOSEPH E. BUMGARNER, April 10, 2001, MEHLMAN v. PHILIP MORRIS INC.. 2001 April 10. Depositions and Trial Testimony (DATTA). Available from: <https://www.industrydocuments.ucsf.edu/docs/jffg0021> Accessed 08 November 2019  Cited in:  Proctor, R. N. (2011). Golden holocaust: Origins of the cigarette catastrophe and the case for abolition. Berkeley: University of California Press. |
| 5 | Contribute Misleading Literature | X | Thompson Carl. Memorandum to Kloepfer W Jr. (Tobacco Institute). Subject: Tobacco and Health Research Procedural Memo. 1968 October 18. Tobacco Institute Records. Available from: <https://www.industrydocuments.ucsf.edu/docs/ymgx0146> Document ID: ymgx0146. Accessed 10 January 2020. |
| 6 | Host Conferences or Seminars | X | Unknown Author. Untitled. 1991 January. Philip Morris Records; Congressman Bliley Philip Morris Collection. Available from: <https://www.industrydocuments.ucsf.edu/docs/yzly0086>. Accessed 08 January 2020. |
| 7 | Avoid/Abuse Peer-Review | X | Review, funded by Tobacco companies, published in a Tobacco-created journal: Thornton, A. J., & Lee, P. N. (1998). Parental smoking and risk of childhood cancer: a review of the evidence. *Indoor and Built Environment*, *7*(2), 65-86.  Evidence that the journal was created by the Tobacco industry:  Garne, D., Watson, M., Chapman, S., & Byrne, F. (2005). Environmental tobacco smoke research published in the journal Indoor and Built Environment and associations with the tobacco industry. *The Lancet*, *365*(9461), 804-809. |
| 8 | Employ Hyperbolic Language | X | Unknown Author. BAD SCIENCE, A RESOURCE BOOK. 1993 March 26. Philip Morris Records. Available from: <https://www.industrydocuments.ucsf.edu/docs/qmcj0065>. Accessed 30 October 2019.  Cited in:  Oreskes, N., & Conway, E. M. (2010). *Merchants of doubt: How a handful of scientists obscured the truth on issues from tobacco smoke to global warming*. New York: Bloomsbury Press. |
| 9 | Blame Other Causes | X | MACKINTOSH BA, WOMBLE CARLYLE. SMOKING AND HEALTH LITIGATION INTEGRATED EXPOSURE AND HAZARD ASSESSMENT INITIATIVE. DESCRIPTIVE PROJECT PROPOSAL AND WORKING NOTES. 1987 January 20. RJ Reynolds Records; Congressman Bliley Philip Morris Collection. Cited in: <https://www.industrydocuments.ucsf.edu/docs/tjpb0024>. Accessed 05 November 2019.  Cited in:  Michaels, D. (2008). *Doubt is their product: How industry's assault on science threatens your health.* Oxford; New York: Oxford University Press, 2008. |
| 10 | Invoke Liberties/Censorship/  Overregulation | X | Author Unknown. FREEDOM ORGANISATION FOR THE RIGHT T. CONFERENCE & RESEARCH PROPOSAL. 1997. RJ Reynolds Records. Available from: https://www.industrydocuments.ucsf.edu/docs/gxpx0094. Accessed 09 January 2020.  Cited in:  Oreskes, N., & Conway, E. M. (2010). *Merchants of doubt: How a handful of scientists obscured the truth on issues from tobacco smoke to global warming*. New York: Bloomsbury Press. Pg 163 |
| 11 | Define How to Measure Outcome/Exposure | X | Unknown Author. FORCE FIELD ANALYSIS. 1997 May. Philip Morris Records. Available from: <https://www.industrydocuments.ucsf.edu/docs/fyyc0069>. Accessed 06 January 2020. |
| 12 | Take Advantage of Scientific Illiteracy | X | BROWN & WILLIAMSON. TRANSCRIPT OF E-CHAT WITH DR. SHARON BOYSE, DIRECTOR OF SCIENTIFIC ISSUES, B&W 20000612 8:00 P.M. EDT. 2000. Philip Morris Records. Available from: <https://www.industrydocuments.ucsf.edu/docs/qtbj0066>. Accessed 28 November 2019.  Michaels, D. (2008). *Doubt is their product: How industry's assault on science threatens your health.* Oxford; New York: Oxford University Press, 2008. Pg 6-7 |
| 13 | Pose as a Defender of Health or Truth | X | BURLEY AUCTION WAREHOUSE ASSOCIATION. A FRANK STATEMENT TO CIGARETTE SMOKERS; R582. 1954. Philip Morris Records. Available from: <https://www.industrydocuments.ucsf.edu/docs/ltln0082>. Accessed 10 January 2020. |
| 14 | Obscure involvement | X | Unknown Author. Confidential - Public Relations in the Field of Smoking and Health. 1963 January. Ness Motley Law Firm Documents. Available from: <https://www.industrydocuments.ucsf.edu/docs/hqky0042>. Accessed 08 December 2019.  Proctor, R. N. (2011). Golden holocaust: Origins of the cigarette catastrophe and the case for abolition. Berkeley: University of California Press. Pg 262 |
| 15 | Develop a PR Strategy | X | Unknown Author. Untitled. 1991 January. Philip Morris Records; Congressman Bliley Philip Morris Collection. Available from: <https://www.industrydocuments.ucsf.edu/docs/yzly0086>. Accessed 08 January 2020. |
| 16 | Appeal to Mass Media | X | COMMITTEE ON ENERGY + COMMERCE, HOUSE, SUBCOMMITTEE ON HEALTH + THE ENVIRONMENT. THE HILL AND KNOWLTON DOCUMENTS: HOW THE TOBACCO INDUSTRY LAUNCHED ITS DISINFORMATION CAMPAIGN. 1995 May 26. Philip Morris Records. Available from: <https://www.industrydocuments.ucsf.edu/docs/krjp0058> Accessed 08 January 2020. |
| 17 | Take Advantage of Victim’s Lack of  Money/Influence |  |  |
| 18 | Normalization | X | BROWN & WILLIAMSON. TRANSCRIPT OF E-CHAT WITH DR. SHARON BOYSE, DIRECTOR OF SCIENTIFIC ISSUES, B&W 20000612 8:00 P.M. EDT. 2000. Philip Morris Records. Available from: <https://www.industrydocuments.ucsf.edu/docs/qtbj0066>. Accessed 28 November 2019. See page 4. |
| 19 | Impede Government Regulation | X | DREYER, LP. WASH TECH CONFERENCE CALL; HANDWRITTEN NOTES OF PHILIP MORRIS IN-HOUSE MEMORIALIZING MEETING BETWEEN PHILIP MORRIS IN-HOUSE COUNSEL AND PHILIP MORRIS REGULATORY CONSULTANTS REGARDING PROPOSED OSHA RULEMAKING. 1994 December 04. Philip Morris Records; Congressman Bliley Philip Morris Collection. Available from: <https://www.industrydocuments.ucsf.edu/docs/rtwv0003> Accessed 06 January 2020.  Cited in:  Michaels, D. (2008). *Doubt is their product: How industry's assault on science threatens your health.* Oxford; New York: Oxford University Press, 2008. |
| 20 | Alter Product to Seem Healthier | X | BROWN & WILLIAMSON. TRANSCRIPT OF E-CHAT WITH DR. SHARON BOYSE, DIRECTOR OF SCIENTIFIC ISSUES, B&W 20000612 8:00 P.M. EDT. 2000. Philip Morris Records. Available from: <https://www.industrydocuments.ucsf.edu/docs/qtbj0066>. Accessed 28 November 2019.  Cited in:  Glantz, S. A. (1996). *The cigarette papers.* Berkeley: University of California Press;  Also cited in:  Oreskes, N., & Conway, E. M. (2010). *Merchants of doubt: How a handful of scientists obscured the truth on issues from tobacco smoke to global warming*. New York: Bloomsbury Press. |
| 21 | Influence Government/Laws | X | SRIC INNOVATION. SOUND SCIENCE PROJECT. 1997 May. Philip Morris Records. Available from: <https://www.industrydocuments.ucsf.edu/docs/snyc0069>. Accessed 08 January 2020. See pg 4 |
| 22 | Attack Opponents |  |  |
| 23 | Appeal to Emotion | X | SRIC INNOVATION. SOUND SCIENCE PROJECT. 1997 May. Philip Morris Records. Available from: <https://www.industrydocuments.ucsf.edu/docs/snyc0069>. Accessed 08 January 2020. |
| 24 | Inappropriately Question Causality | X | Brown & Williamson 1967, discussed in: Bates, C., & Rowell, A. (2004). Tobacco Explained... The truth about the tobacco industry... in its own words. Available from: <https://www.who.int/tobacco/media/en/TobaccoExplained.pdf>. Accessed 04 November 2019.  Also discussed at length in:  Milberger S, Davis RM, Douglas CE, Beasley JK, Burns D, Houston T, Shopland D. Tobacco manufacturers' defence against plaintiffs' claims of cancer causation: throwing mud at the wall and hoping some of it will stick. Tob Control 2006; 15 Suppl 4:iv17-iv26 |
| 25 | Straw Man Arguments |  |  |
| 26 | Abusing Credentials | X | Discussed in: Oreskes, N., & Conway, E. M. (2010). *Merchants of doubt: How a handful of scientists obscured the truth on issues from tobacco smoke to global warming*. New York: Bloomsbury Press. See Pgs 5-6. |
| 27 | Abuse Date Access Requests | X | SRIC INNOVATION. SOUND SCIENCE PROJECT. 1997 May. Philip Morris Records. Available from: <https://www.industrydocuments.ucsf.edu/docs/snyc0069>. Accessed 08 January 2020. See pg 3  Also discussed in:  Michaels, D. (2008). *Doubt is their product: How industry's assault on science threatens your health.* Oxford; New York: Oxford University Press, 2008. See pg 177. |
| 28 | Slippery Slope | X | JEFFREYS K, SINGER SF. THE EPA AND THE SCIENCE OF ENVIRONMENTAL TOBACCO SMOKE. 1994 May. Tobacco Institute Records. Available from: <https://www.industrydocuments.ucsf.edu/docs/tzbc0065>. Accessed 06 January 2020. |

Supplemental Table 2. List of Strategies Used by the Coal Industry and Affiliates

| # | Strategy | X = used | Evidence Available From: |
| --- | --- | --- | --- |
| 1 | Attack Study Design | X | Hamby, C. (2013). As experts recognize new form of black lung, coal industry follows familiar pattern of denial. In *Breathless and Burdened* (3). Available from https://www.publicintegrity.org/2013/11/01/13653/experts-recognize-new-form-black-lung-coal-industry-follows-familiar-pattern-denial. Accessed 01 March 2018. See pg 11  Fino GJ, Bahl BJ. Proposed changes to the regulations implementing the Federal Black Lung Benefits Act. 1999. Available from: <https://www.documentcloud.org/documents/813278-fino-report-for-national-mining-association.html> Accessed 10 January 2020 |
| 2 | Gain Support from Reputable Individuals | X | Hamby, C. (2013). Johns Hopkins medical unit rarely finds black lung, helping coal industry defeat miners' claims. In *Breathless and Burdened* (2). Available from <https://www.publicintegrity.org/2013/10/30/13637/johns-hopkins-medical-unit-rarely-finds-black-lung-helping-coal-industry-defeat> Accessed 01 March 2018. See pg 2 |
| 3 | Misrepresent Data | X | - Hamby, C. (2013). Coal industry’s go-to law firm withheld evidence of black lung, at expense of sick miners. In *Breathless and Burdened* (1). Available from https://www.publicintegrity.org/2013/10/29/13585/coal-industrys-go-law-firm-withheld-evidence-black-lung-expense-sick-miners. Accessed 01 March 2018. See pg 9 |
| 4 | Suppress Incriminating Information | X | Hamby, C. (2013). Coal industry’s go-to law firm withheld evidence of black lung, at expense of sick miners. In *Breathless and Burdened* (1). Available from https://www.publicintegrity.org/2013/10/29/13585/coal-industrys-go-law-firm-withheld-evidence-black-lung-expense-sick-miners. Accessed 01 March 2018. See pgs 8 and 19 |
| 5 | Contribute Misleading Literature |  |  |
| 6 | Host Conferences or Seminars |  |  |
| 7 | Avoid/Abuse Peer-Review |  |  |
| 8 | Employ Hyperbolic Language | X | Hamby, C. (2013). Johns Hopkins medical unit rarely finds black lung, helping coal industry defeat miners' claims. In *Breathless and Burdened* (2). Available from <https://www.publicintegrity.org/2013/10/30/13637/johns-hopkins-medical-unit-rarely-finds-black-lung-helping-coal-industry-defeat> Accessed 01 March 2018. See pg 6 |
| 9 | Blame Other Causes | X | Smith, B. E. (1981). Black lung: The social production of disease. *International Journal of Health Services, 11*(3), 343-359. |
| 10 | Invoke Liberties/Censorship/  Overregulation |  |  |
| 11 | Define How to Measure Outcome/Exposure | X | Hamby, C. (2013). Johns Hopkins medical unit rarely finds black lung, helping coal industry defeat miners' claims. In *Breathless and Burdened* (2). Available from <https://www.publicintegrity.org/2013/10/30/13637/johns-hopkins-medical-unit-rarely-finds-black-lung-helping-coal-industry-defeat> Accessed 01 March 2018. See pgs 3 and 16 |
| 12 | Take Advantage of Scientific Illiteracy |  |  |
| 13 | Pose as a Defender of Health or Truth |  |  |
| 14 | Obscure involvement |  |  |
| 15 | Develop a PR Strategy |  |  |
| 16 | Appeal to Mass Media |  |  |
| 17 | Take Advantage of Victim’s Lack of  Money/Influence | X | Hamby, C. (2013). Coal industry’s go-to law firm withheld evidence of black lung, at expense of sick miners. In *Breathless and Burdened* (1). Available from https://www.publicintegrity.org/2013/10/29/13585/coal-industrys-go-law-firm-withheld-evidence-black-lung-expense-sick-miners. Accessed 01 March 2018. See pgs 2 and 19 |
| 18 | Normalization | X | Smith, B. E. (1981). Black lung: The social production of disease. *International Journal of Health Services, 11*(3), 343-359. |
| 19 | Impede Government Regulation |  |  |
| 20 | Alter Product to Seem Healthier |  |  |
| 21 | Influence Government/Laws | X | Hamby, C. (2013). As experts recognize new form of black lung, coal industry follows familiar pattern of denial. In *Breathless and Burdened* (3). Available from https://www.publicintegrity.org/2013/11/01/13653/experts-recognize-new-form-black-lung-coal-industry-follows-familiar-pattern-denial. Accessed 01 March 2018. See pgs 11 and 13  Murray RE. Murray Energy’s ‘Action Plan’ for the Trump Administration. Available from:  https://www.nytimes.com/interactive/2018/01/09/climate/document-Murray-Energy-Action-Plan.html  Accessed 03 January 2020. |
| 22 | Attack Opponents |  |  |
| 23 | Appeal to Emotion |  |  |
| 24 | Inappropriately Question Causality |  |  |
| 25 | Straw Man Arguments |  |  |
| 26 | Abusing Credentials |  |  |
| 27 | Abuse Date Access Requests |  |  |
| 28 | Slippery Slope |  |  |

Supplemental Table 3. List of Strategies Used by the Sugar Industry and its Affiliates

| # | Strategy | X = used | Evidence Available From: |
| --- | --- | --- | --- |
| 1 | Attack Study Design | X | Kearns, C. E., Schmidt, L. A., & Glantz, S. A. (2016). Sugar industry and coronary heart disease research: A historical analysis of internal industry documents. *Jama Internal Medicine, 176*(11), 1680-1685. |
| 2 | Gain Support from Reputable Individuals | X | O’Connor, A. (2016, September 12). How the Sugar Industry Shifted Blame to Fat. *New York Times*. Retrieved from <https://www.nytimes.com/2016/09/13/well/eat/how-the-sugar-industry-shifted-blame-to-fat.html>. Accessed 05 December 2019. |
| 3 | Misrepresent Data | X | - Kearns, C. E., Schmidt, L. A., & Glantz, S. A. (2016). Sugar industry and coronary heart disease research: A historical analysis of internal industry documents. *Jama Internal Medicine, 176*(11), 1680-1685. - Hegsted D.M., McGandy R.B., Myers M.L., & Stare F.J. (1965). Quantitative effects of dietary fat on serum cholesterol in man. *American Journal of Clinical Nutrition, 17*(5), 281-295. |
| 4 | Suppress Incriminating Information | X | Kearns, C. E., Apollonio, D., & Glantz, S. A. (2017). Sugar industry sponsorship of germ-free rodent studies linking sucrose to hyperlipidemia and cancer: An historical analysis of internal documents. *PLoS Biology, 15*(11), 1-9. |
| 5 | Contribute Misleading Literature | X | "United States: Sugar Papers Reveal Industry Role in Shifting National Heart Disease Focus to Saturated Fat." Mena Report, 14 Sept. 2016. *Gale Academic Onefile.* Available from: <https://link.gale.com/apps/doc/A467421745/AONE?u=9211haea&sid=AONE&xid=6633c9e7>. Accessed 08 January 2020. |
| 6 | Host Conferences or Seminars | X | Kearns, C. E., Schmidt, L. A., & Glantz, S. A. (2016). Sugar industry and coronary heart disease research: A historical analysis of internal industry documents. *JAMA Internal Medicine, 176*(11), 1680-1685. Retrieved from <https://jamanetwork.com/journals/jamainternalmedicine/fullarticle/2548255>.  [Kearns et al. cites this document, which is not available at University of Illinois archives based on this citation]: Hickson JL. Memoranda to Neil Kelly regarding possible activities of the Sugar Association Inc (December 14, 1964). Papers of Roger Adams at the University of Illinois Archives, 1889-1971. Urbana: University of Illinois. Record Series No. 15/5/23.  Stewart SS. A research profile of sugar: as a food, as a chemical raw material; Seventh International Sugar Research Symposium, London, England, September 12 - 13, 1973. Bethesda: International Sugar Research Foundation; 1974. p. 57.  Cited in:  Kearns CE, Glantz SA, Apollonio DE. (2019). In defense of sugar: a critical analysis of rhetorical strategies used in The Sugar Association’s award-winning 1976 public relations campaign. *BMC Public Health*. 19(1), 1150. |
| 7 | Avoid/Abuse Peer-Review | X | O’Connor, A. (2016, September 12). How the Sugar Industry Shifted Blame to Fat. *New York Times*. Retrieved from <https://www.nytimes.com/2016/09/13/well/eat/how-the-sugar-industry-shifted-blame-to-fat.html>. Accessed 05 December 2019. |
| 8 | Employ Hyperbolic Language | X | Kearns, C. E., Schmidt, L. A., & Glantz, S. A. (2016). Sugar industry and coronary heart disease research: A historical analysis of internal industry documents. *JAMA Internal Medicine, 176*(11), 1680-1685.  McGandy RB, Hegsted DM, Stare FJ.  Dietary fats, carbohydrates and atherosclerotic vascular disease. *N Engl J Med*. 1967;277(5):245-247.  The Sugar Association Inc. The Sugar Association Inc. and Carl Byoir & Associates, Inc. Public Relations Society of America Records. Madison, WI: Wisconsin Historical Society; 1976.  Cited in:  Kearns CE, Glantz SA, Apollonio DE. (2019). In defense of sugar: a critical analysis of rhetorical strategies used in The Sugar Association’s award-winning 1976 public relations campaign. *BMC Public Health*. 19(1), 1150. |
| 9 | Blame Other Causes | X | McGandy RB, Hegsted DM, Stare FJ.  Dietary fats, carbohydrates and atherosclerotic vascular disease. *N Engl J Med*. 1967;277(5):245-247. |
| 10 | Invoke Liberties/Censorship/  Overregulation |  |  |
| 11 | Define How to Measure Outcome/Exposure | X | Kearns, C. E., Schmidt, L. A., & Glantz, S. A. (2016). Sugar industry and coronary heart disease research: A historical analysis of internal industry documents. *JAMA Internal Medicine, 176*(11), 1680-1685. |
| 12 | Take Advantage of Scientific Illiteracy | X | Coping with new dangers: an action plan for Sugar Information in the face of mounting criticism, public relations proposals for 1971–1972. William Jefferson Darby Papers. Nashville: Eskind Biomedical LIbrary Special Collections, Vanderbilt University Medical Center; 1971.  Cited in:  Kearns CE, Glantz SA, Apollonio DE. (2019). In defense of sugar: a critical analysis of rhetorical strategies used in The Sugar Association’s award-winning 1976 public relations campaign. *BMC Public Health*. 19(1), 1150. |
| 13 | Pose as a Defender of Health or Truth | X | Kearns, C. E., Schmidt, L. A., & Glantz, S. A. (2016). Sugar industry and coronary heart disease research: A historical analysis of internal industry documents. *JAMA Internal Medicine, 176*(11), 1680-1685. |
| 14 | Obscure involvement | X | O’Connor, A. (2016, September 12). How the Sugar Industry Shifted Blame to Fat. *New York Times*. Retrieved from <https://www.nytimes.com/2016/09/13/well/eat/how-the-sugar-industry-shifted-blame-to-fat.html>. Accessed 05 December 2019. |
| 15 | Develop a PR Strategy | X | Kearns, C. E., Schmidt, L. A., & Glantz, S. A. (2016). Sugar industry and coronary heart disease research: A historical analysis of internal industry documents. *JAMA Internal Medicine, 176*(11), 1680-1685.  [Kearns et al. cites this document, which is not available at University of Illinois archives based on this citation]: Hickson, J.L. (1964). Memoranda to Neil Kelly regarding possible activities of the Sugar Association Inc. Papers of Roger Adams at the University of Illinois Archives, 1889–1971. Record Series No. 15/5/23. |
| 16 | Appeal to Mass Media |  |  |
| 17 | Take Advantage of Victim’s Lack of  Money/Influence |  |  |
| 18 | Normalization |  |  |
| 19 | Impede Government Regulation |  |  |
| 20 | Alter Product to Seem Healthier |  |  |
| 21 | Influence Government/Laws | X | Kearns, C. E., Schmidt, L. A., & Glantz, S. A. (2016). Sugar industry and coronary heart disease research: A historical analysis of internal industry documents. *JAMA Internal Medicine, 176*(11), 1680-1685.  Taubes G. & Couzens C.K (2012). Big sugar’s sweet little lies: how the industry kept scientists from asking, does sugar kill? Available from: <http://www.motherjones.com/environment/2012/10/sugar-industry-lies-campaign>. Accessed 10 April 2018.  Kearns, C. E., Glantz, S. A., & Schmidt, L. A. (2015). Sugar industry influence on the scientific agenda of the National Institute of Dental Research’s 1971 National Caries Program: a historical analysis of internal documents. *PLoS medicine*, *12*(3), e1001798.  Sugar Association (2015). *Sugar and heart health: What are the facts?*. Retrieved from <https://www.sugar.org/sugar-heart-health-facts/> Accessed 04 March 2018. [website no longer available in Jan 2020]  Briscoe AC, Gaine PC. Sugar Association Comment on the 2015 DGAC Report. Available from: <https://health.gov/dietaryguidelines/dga2015/comments/readCommentDetails.aspx?CID=22978>. Accessed 12 January 2020. |
| 22 | Attack Opponents |  |  |
| 23 | Appeal to Emotion |  |  |
| 24 | Inappropriately Question Causality |  |  |
| 25 | Straw Man Arguments |  |  |
| 26 | Abusing Credentials |  |  |
| 27 | Abuse Date Access Requests |  |  |
| 28 | Slippery Slope |  |  |

Supplemental Table 4. List of Strategies used by Syngenta Related to Atrazine

| # | Strategy | X = used | Evidence Available From: |
| --- | --- | --- | --- |
| 1 | Attack Study Design | X | Aviv, R. (2014, Feb 10). A valuable reputation. *New York Times.* Retrieved from https://www.newyorker.com/magazine/2014/02/10/a-valuable-reputation. Accessed 21 December 2018. See Exhibit 19, pg 4. |
| 2 | Gain Support from Reputable Individuals | X | Unknown Author. Exhibit 11 - *Supportive Third Party Stakeholder Database.* Email Correspondence. Available from <https://assets.documentcloud.org/documents/686401/100reporters-syngenta-clare-howard-investigation.pdf>. Accessed 21 December 2018. |
| 3 | Misrepresent Data | X | - Hayes TB. (2004). There Is No Denying This: Defusing the Confusion about Atrazine. BioScience, 54(12), 1138. |
| 4 | Suppress Incriminating Information | X | Kay C. Exhibit 11 - *Pro-Active Ideas.* Email Correspondence. Available from <https://assets.documentcloud.org/documents/686401/100reporters-syngenta-clare-howard-investigation.pdf>. Accessed 21 December 2018.  Unknown Author. Exhibit 13 - *White House Writers Group. Syngenta Crop Protection PR/ Advertising Agency RFP September 2009*. Available from <https://assets.documentcloud.org/documents/686402/100reporters-syngenta-clare-howard-investigation.pdf>, Accessed 18 November 2018. See pgs 11 and 24 |
| 5 | Contribute Misleading Literature | X | Carr J, et al. (2003). Response of larval Xenopus laevis to atrazine: Assessment of growth, metamorphosis, and gonadal and laryngeal morphology. *Environmental Toxicology and Chemistry 22,* 396–405.  Hayes TB. (2004). There Is No Denying This: Defusing the Confusion about Atrazine. BioScience, 54(12), 1138. |
| 6 | Host Conferences or Seminars | X | Unknown Author. Exhibit 13 - *White House Writers Group. Syngenta Crop Protection PR/ Advertising Agency RFP September 2009*. Available from <https://assets.documentcloud.org/documents/686402/100reporters-syngenta-clare-howard-investigation.pdf>, Accessed 18 November 2018. See pgs 6 and 24 |
| 7 | Avoid/Abuse Peer-Review |  |  |
| 8 | Employ Hyperbolic Language | X | Unknown author. Exhibit 07 - *Jayne Thompson Testimonial.* Email Correspondence. Available from <https://assets.documentcloud.org/documents/686398/100reporters-syngenta-clare-howard-investigation.pdf>, Accessed 26 November 2018. See pg 6 |
| 9 | Blame Other Causes |  |  |
| 10 | Invoke Liberties/Censorship/  Overregulation |  |  |
| 11 | Define How to Measure Outcome/Exposure | X | Unknown Author. Exhibit 13 - *White House Writers Group. Syngenta Crop Protection PR/ Advertising Agency RFP September 2009*. Available from <https://assets.documentcloud.org/documents/686402/100reporters-syngenta-clare-howard-investigation.pdf>, Accessed 18 November 2018. See pgs 6-18 |
| 12 | Take Advantage of Scientific Illiteracy | X | Unknown Author. Exhibit 13 - *White House Writers Group. Syngenta Crop Protection PR/ Advertising Agency RFP September 2009*. Available from <https://assets.documentcloud.org/documents/686402/100reporters-syngenta-clare-howard-investigation.pdf>, Accessed 18 November 2018. See pg 8 |
| 13 | Pose as a Defender of Health or Truth | X | Unknown Author. Exhibit 13 - *White House Writers Group. Syngenta Crop Protection PR/ Advertising Agency RFP September 2009*. Available from <https://assets.documentcloud.org/documents/686402/100reporters-syngenta-clare-howard-investigation.pdf>, Accessed 18 November 2018. See pg 8 |
| 14 | Obscure involvement | X | Unknown author. Exhibit 07 - *Jayne Thompson Testimonial.* Email Correspondence. Available from <https://assets.documentcloud.org/documents/686398/100reporters-syngenta-clare-howard-investigation.pdf>, Accessed 26 November 2018. See pg 12 |
| 15 | Develop a PR Strategy | X | Exhibit 11 - *Pro-Active Ideas.* Email Correspondence. Retrieved from https://assets.documentcloud.org/documents/686401/100reporters-syngenta-clare-howard-investigation.pdf. |
| 16 | Appeal to Mass Media | X | Unknown Author. Exhibit 13 - *White House Writers Group. Syngenta Crop Protection PR/ Advertising Agency RFP September 2009*. Available from <https://assets.documentcloud.org/documents/686402/100reporters-syngenta-clare-howard-investigation.pdf>, Accessed 18 November 2018. See pgs 9-10 |
| 17 | Take Advantage of Victim’s Lack of  Money/Influence |  |  |
| 18 | Normalization |  |  |
| 19 | Impede Government Regulation | X | Unknown Author. Exhibit 13 - *White House Writers Group. Syngenta Crop Protection PR/ Advertising Agency RFP September 2009*. Available from <https://assets.documentcloud.org/documents/686402/100reporters-syngenta-clare-howard-investigation.pdf>, Accessed 18 November 2018. See pg 2. |
| 20 | Alter Product to Seem Healthier |  |  |
| 21 | Influence Government/Laws | X | Unknown Author. Exhibit 13 - *White House Writers Group. Syngenta Crop Protection PR/ Advertising Agency RFP September 2009*. Available from <https://assets.documentcloud.org/documents/686402/100reporters-syngenta-clare-howard-investigation.pdf>, Accessed 18 November 2018. |
| 22 | Attack Opponents | X | Ford SD. Exhibit 19 - *Sherry Duvall Ford*. Notes in Diary. Available from <https://assets.documentcloud.org/documents/686406/100reporters-syngenta-clare-howard-investigation.pdf>. Accessed 08 December 2018. |
| 23 | Appeal to Emotion | X | Unknown Author. Exhibit 13 - *White House Writers Group. Syngenta Crop Protection PR/ Advertising Agency RFP September 2009*. Available from <https://assets.documentcloud.org/documents/686402/100reporters-syngenta-clare-howard-investigation.pdf>, Accessed 18 November 2018. See pg 4. |
| 24 | Inappropriately Question Causality |  |  |
| 25 | Straw Man Arguments |  |  |
| 26 | Abusing Credentials |  |  |
| 27 | Abuse Date Access Requests | X | Ford SD. Exhibit 19 - *Sherry Duvall Ford*. Notes in Diary. Available from <https://assets.documentcloud.org/documents/686406/100reporters-syngenta-clare-howard-investigation.pdf>. Accessed 08 December 2018. See pg 5 |
| 28 | Slippery Slope | X | Aviv, R. (2014, Feb 10). A valuable reputation. *New York Times.* Retrieved from https://www.newyorker.com/magazine/2014/02/10/a-valuable-reputation. Accessed 21 December 2018. See Exhibit 13, pg 16. |

Supplemental Table 5. List of Strategies Used by Scientists in and near the Marshall Institute in the 1980s

| # | Strategy | X = used | Evidence Available From: |
| --- | --- | --- | --- |
| 1 | Attack Study Design | X | US House of Representatives Committee on Science. (1995). Hearing on Climate Models and Projections of Potential Impacts of Global Climate Change. Available from: <https://archive.org/details/scientificintegr111695unit/page/1076>. Accessed 13 January 2020. See pg 1076.  Cited in:  Oreskes, N., & Conway, E. M. (2010). *Merchants of doubt: How a handful of scientists obscured the truth on issues from tobacco smoke to global warming*. |
| 2 | Gain Support from Reputable Individuals | X | Oreskes, N., & Conway, E. M. (2010). *Merchants of doubt: How a handful of scientists obscured the truth on issues from tobacco smoke to global warming*. New York: Bloomsbury Press. See pg 190. |
| 3 | Misrepresent Data | X | Jastrow, R., Nierenberg, W., & Seitz, F. (1991). Global warming: What does the science tell us?. *Energy*, *16*(11-12), 1331-1345.  Hansen, J., Johnson, D., Lacis, A., Lebedeff, S., Lee, P., Rind, D., & Russell, G. (1981). Climate Impact of Increasing Atmospheric Carbon Dioxide.  *Science,* *213*(4511), 957-966.  Oreskes, N., & Conway, E. M. (2010). *Merchants of doubt: How a handful of scientists obscured the truth on issues from tobacco smoke to global warming*. |
| 4 | Suppress Incriminating Information |  |  |
| 5 | Contribute Misleading Literature | X | Jastrow, R., Nierenberg, W., & Seitz, F. (1991). Global warming: What does the science tell us?. *Energy*, *16*(11-12), 1331-1345.  National Research Council, & Carbon Dioxide Assessment Committee. (1983). *Changing climate: Report of the carbon dioxide assessment committee*. National Academies. See Chapter 2. |
| 6 | Host Conferences or Seminars |  |  |
| 7 | Avoid/Abuse Peer-Review | X | Singer, S. (1996). Climate Change and Consensus. *Science,271*(5249), 581-582. See pg 582  Wigley, T., & Singer, S. (1996). Climate Change Report. *Science,271*(5255), 1481-1483. See pg 1483  Lahsen, M. (2008). Experiences of modernity in the greenhouse: A cultural analysis of a physicist “trio” supporting the backlash against global warming. *Global Environmental Change, 18*(1), 204-219.  Oreskes, N., & Conway, E. M. (2010). *Merchants of doubt: How a handful of scientists obscured the truth on issues from tobacco smoke to global warming*. See pg 244 |
| 8 | Employ Hyperbolic Language | X | Wigley, T., & Singer, S. (1996). Climate Change Report. *Science,271*(5255), 1481-1483.  Oreskes, N., & Conway, E. M. (2010). *Merchants of doubt: How a handful of scientists obscured the truth on issues from tobacco smoke to global warming*. See pg 206 |
| 9 | Blame Other Causes | X | Jastrow, R., Nierenberg, W., & Seitz, F. (1991). Global warming: What does the science tell us?. *Energy*, *16*(11-12), 1331-1345.  Hansen, J., Johnson, D., Lacis, A., Lebedeff, S., Lee, P., Rind, D., & Russell, G. (1981). Climate Impact of Increasing Atmospheric Carbon Dioxide. *Science,* *213*(4511), 957-966.  Oreskes, N., & Conway, E. M. (2010). *Merchants of doubt: How a handful of scientists obscured the truth on issues from tobacco smoke to global warming*. |
| 10 | Invoke Liberties/Censorship/  Overregulation | X | Lahsen, M. (1999). The detection and attribution of conspiracies: the controversy over Chapter 8. *Paranoia within reason: A casebook on conspiracy as explanation*, *6*, 111-136. See pg 128  Oreskes, N., & Conway, E. M. (2010). *Merchants of doubt: How a handful of scientists obscured the truth on issues from tobacco smoke to global warming*. See pg 214 |
| 11 | Define How to Measure Outcome/Exposure |  |  |
| 12 | Take Advantage of Scientific Illiteracy | X | Oreskes, N., & Conway, E. M. (2010). *Merchants of doubt: How a handful of scientists obscured the truth on issues from tobacco smoke to global warming*. See pg 202  Stevens, W. K. (1995 Sep 10). Experts call human role likely. *The New York Times*, *10*. Available from: <https://www.nytimes.com/1995/09/10/world/global-warming-experts-call-human-role-likely.html>. Accessed 11 January 2020. |
| 13 | Pose as a Defender of Health or Truth | X | Seitz, F. (1996 June 12). A major deception on global warming. *Wall Street Journal*, *12*. Available from: <https://www.wsj.com/articles/SB834512411338954000>. Accessed 08 January 2020.  Oreskes, N., & Conway, E. M. (2010). *Merchants of doubt: How a handful of scientists obscured the truth on issues from tobacco smoke to global warming*. See pg. 208 |
| 14 | Obscure involvement |  |  |
| 15 | Develop a PR Strategy |  |  |
| 16 | Appeal to Mass Media | X | Oreskes, N., & Conway, E. M. (2010). *Merchants of doubt: How a handful of scientists obscured the truth on issues from tobacco smoke to global warming*. See pg 214  Linden, E. (2006). *The winds of change: Climate, weather, and the destruction of civilizations*. Simon and Schuster. See pgs. 222-23. |
| 17 | Take Advantage of Victim’s Lack of  Money/Influence | X | Nunley, J. (Interviewer) & Barron, D. (Interviewee). (1994). *Global Warming Lawsuit* [Interview transcript]. Living on Earth. Transcript available from: <https://www.loe.org/shows/segments.html?programID=94-P13-00008&segmentID=1>. Accessed 13 January 2020.  Oreskes, N., & Conway, E. M. (2010). *Merchants of doubt: How a handful of scientists obscured the truth on issues from tobacco smoke to global warming*. |
| 18 | Normalization | X | National Research Council, & Carbon Dioxide Assessment Committee. (1983). *Changing climate: Report of the carbon dioxide assessment committee*. National Academies. See Chapter 2, pg 3.  Oreskes, N., & Conway, E. M. (2010). *Merchants of doubt: How a handful of scientists obscured the truth on issues from tobacco smoke to global warming*. |
| 19 | Impede Government Regulation |  |  |
| 20 | Alter Product to Seem Healthier |  |  |
| 21 | Influence Government/Laws | X | Oreskes, N., & Conway, E. M. (2010). *Merchants of doubt: How a handful of scientists obscured the truth on issues from tobacco smoke to global warming*. See pg 190  Robert Jastrow to Terry Yosle, (1991, February 2). *WAN papers*. Accession 2001-01, 60: file label “Marshall Institute Correspondence, 1990-1992,” SIO Archives. |
| 22 | Attack Opponents | X | Lahsen, M. (1999). The detection and attribution of conspiracies: the controversy over Chapter 8. *Paranoia within reason: A casebook on conspiracy as explanation*, *6*, 111-136. See pg 127. |
| 23 | Appeal to Emotion |  |  |
| 24 | Inappropriately Question Causality |  |  |
| 25 | Straw Man Arguments | X | Wigley, T., & Singer, S. (1996). Climate Change Report. *Science,271*(5255), 1481-1483.  Oreskes, N., & Conway, E. M. (2010). *Merchants of doubt: How a handful of scientists obscured the truth on issues from tobacco smoke to global warming*. See pg 206 |
| 26 | Abusing Credentials | X | Oreskes, N., & Conway, E. M. (2010). *Merchants of doubt: How a handful of scientists obscured the truth on issues from tobacco smoke to global warming*. See pg 271 |
| 27 | Abuse Date Access Requests |  |  |
| 28 | Slippery Slope |  |  |
